# High and uneven levels of 45S rDNA site-number variation across wild populations of a diploid plant genus (*Anacyclus*, Asteraceae)

**DOI:** 10.1371/journal.pone.0187131

**Published:** 2017-10-31

**Authors:** Marcela Rosato, Inés Álvarez, Gonzalo Nieto Feliner, Josep A. Rosselló

**Affiliations:** 1 Jardín Botánico, ICBIBE-Unidad Asociada CSIC, Universidad de Valencia, Valencia, Spain; 2 Real Jardín Botánico (CSIC), Madrid, Spain; Università di Pisa, ITALY

## Abstract

The nuclear genome harbours hundreds to several thousand copies of ribosomal DNA. Despite their essential role in cellular ribogenesis few studies have addressed intrapopulation, interpopulation and interspecific levels of rDNA variability in wild plants. Some studies have assessed the extent of rDNA variation at the sequence and copy-number level with large sampling in several species. However, comparable studies on rDNA site number variation in plants, assessed with extensive hierarchical sampling at several levels (individuals, populations, species) are lacking. In exploring the possible causes for ribosomal loci dynamism, we have used the diploid genus *Anacyclus* (Asteraceae) as a suitable system to examine the evolution of ribosomal loci. To this end, the number and chromosomal position of 45S rDNA sites have been determined in 196 individuals from 47 populations in all *Anacyclus* species using FISH. The 45S rDNA site-number has been assessed in a significant sample of seed plants, which usually exhibit rather consistent features, except for polyploid plants. In contrast, the level of rDNA site-number variation detected in *Anacyclus* is outstanding in the context of angiosperms particularly regarding populations of the same species. The number of 45S rDNA sites ranged from four to 11, accounting for 14 karyological ribosomal phenotypes. Our results are not even across species and geographical areas, and show that there is no clear association between the number of 45S rDNA loci and the life cycle in *Anacyclus*. A single rDNA phenotype was detected in several species, but a more complex pattern that included intra-specific and intra-population polymorphisms was recorded in *A*. *homogamos*, *A*. *clavatus* and *A*. *valentinus*, three weedy species showing large and overlapping distribution ranges. It is likely that part of the cytogenetic changes and inferred dynamism found in these species have been triggered by genomic rearrangements resulting from contemporary hybridisation.

## Introduction

In most plant species, repetitive DNA constitutes a large fraction of the nuclear genome and is a major contributor to plant chromosome structure. The amplification, deletion and transposition of different repeated DNA motifs, and the spread of sequence mutations across the genome produces a turnover of repetitive DNA that has been associated to population divergence and speciation processes [1].

Few coding regions are part of the repetitive DNA with the exception of the ribosomal DNA (rDNA) and the coding genes in transposable elements. The nuclear genome harbors hundreds to several thousand copies of each ribosomal family, 45S (18S, 5.8S, 25S/26S) and 5S, which are usually arranged in distinct arrays of tandemly-repeated units [2]. Despite their essential role in cellular ribogenesis and organismal growth and integrity, few studies have addressed intrapopulation, interpopulation and interspecific levels of rDNA variability in wild plants (i.e., site number and genomic location, copy number, and sequence divergence). There have been studies assessing the extent of rDNA variation at the sequence and copy-number level with large hierarchical sampling in several species [3, 4]. However, comparable studies on rDNA site number variation in plants, assessed with extensive sampling at several levels (individuals, populations, species) are lacking.

Inter-individual fluctuations in the amplification or deletion of rDNA arrays over evolutionary time have been inferred to occur in several plant groups [5–7]. Although it is known that 45S rDNA sites may display intra-specific variation in size and number, most of the variation reported thus far is restricted to artificial hybrids, polyploid systems, and crop species [8–12]. Hence, modifications in their rDNA genome organization may have occurred as a result of intense agronomic selection [10] or the artificial origin of the accessions used (e.g., in the Jemalong J5 and R-1081 lines obtained in the model species *Medicago truncatula*; [8, 12]).

It has been reported that rDNA loci are the predominant targets of repeated recombination events [13]. Thus, illegitimate recombination between loci may trigger both intragenomic variation in rDNA copy number and amplification of new arrays [14, 15]. Furthermore, it has been shown that rDNA arrays and neighboring regions are one of the frequent targets for mobile element insertions [16]. Accordingly, transposition may promote changes in the number of rDNA loci with potential evolutionary consequences, such as variability in the genome size or the generation of reproductive isolation as a consequence of meiotic irregularities due to chromosomal divergences. Ultimately, karyological rearrangements resulting from those transposition events have been reported to be associated to population differentiation and speciation even underlying radiative processes [10]. To assess if changes in rDNA loci can be associated to evolutionary processes such as differentiation and speciation, variation has to be analysed from the intra- to the interspecies level, using suitable case-studies and large sampling schemes. We think that analysing entire genera lacking polyploid species with conventional karyological data may provide relevant data on these issues.

*Anacyclus* (Asteraceae, Anthemideae) is a diploid (2n = 18) Mediterranean genus that includes two perennial and seven annual mostly weedy species. This genus represents a suitable system to examine the evolution of ribosomal loci. The gross morphology of chromosomes assessed using conventional staining suggests very similar karyotypes across species [17]. But the analysis of Giemsa C-banded karyotypes strongly suggested a high incidence of structural heterozygosity, mainly in the annual species [18]. The latter results are in line with meiotic studies in artificial hybrids by Humphries [19], who concluded that evolution in *Anacyclus* was linked to chromosomal patterning involving structural changes.

Assessments of the number of nucleolar organizing regions (NOR), the active sites of 45S rDNA loci, are also available for *Anacyclus*. Consistent results were reported within perennials (two loci) and annuals (three loci; [18, 19], which apparently supports the idea of rDNA stability in this genus. Unfortunately, these results were based on a very limited number of individuals from each species. In addition, the fact that NOR sites represent only a fraction of the 45S rDNA loci in the genome, when other transcriptionally inactive rDNA loci are present, indicates that variation in the number of rDNA sites in *Anacyclus* is insufficiently understood and deserves additional attention with the aid of a more refined methodology.

In exploring the possible causes for ribosomal loci dynamism, *Anacyclus* offers an additional element of interest since hybridisation was reported to be in the origin of some species (*A*. *valentinus*, a putative hybrid species originated from crosses between *A*. *homogamos* and *A*. *radiatus* according to [20]). It is well known that the merging of two genomes of different backgrounds in the same organism, can cause genomic stress. Further, it has been found to trigger structural variation in annual species of *Helianthus* [21].

In this study, we aim to clarify the degree of structural rDNA stability in *Anacyclus* using a hierarchical sampling strategy (species, populations and individuals) to allow exploring if patterns of variation are associated to differentiation and/or to species. To this end, the number and chromosomal position of 45S rDNA loci have been determined in all *Anacyclus* species, using fluorescence *in situ* hybridisation (FISH). Specifically, our aims were to (i) determine the extent of variation of rDNA sites in the karyotype of *Anacyclus* and how it is apportioned (ii) infer possible underlying mechanisms for the rDNA diversity associated to taxonomic boundaries [20, 22], geographic distribution, and biological attributes (e.g., life cycles); and (iii) explore whether differential patterns for rDNA site location and number change are evenly distributed within species.

## Materials and methods

### Plant materials

One hundred and ninety six individuals from 47 populations of nine *Anacyclus* species and one subspecies were analysed in this study (S1 Table). This sampling covers all the species according to a recent molecular phylogenetic analysis (Vitales et al., unpubl. res.) concluding that the genus is integrated by nine species (S1 Table). At least five individuals were sampled for each species. Those species with a wider range and reported to be involved in ongoing hybridisation were more heavily sampled (with up to 12 samples per population).

Seeds were collected in the field, and those with the fastest rate of germination were cultivated under conditions described by [23].

### Cytogenetic analysis

#### Fluorescence *in situ* hybridisation

For mitotic chromosome preparations, the protocols described in Rosato et al. [12] were followed. The 45S rDNA multigene family was localised using the pTa71 [24] clone. The pTa71 probe was labelled with digoxigenin-11dUTP through a nick translation procedure (Roche, Germany). Probe detection was conducted using the method of Zhong et al. [25] with modifications according to Galián et al. [26].

#### Karyotype analysis

Chromosome measurements were made on digital images using the computer application MicroMeasure version 3.2 [27]. Idiograms were obtained from chromosome measurements of at least five well-spread metaphase plates. Ribosomal phenotypes were defined as those karyotypes that differ in site number, chromosomal location, and gene dosage (i.e, homozygotic vs. heterozygotic).

#### Evolutionary analysis of rDNA site-number variation

In order to explore the possible patterns of cytogenetic evolution in *Anacyclus*, the results were integrated in a network that visualizes the diversity and frequency of rDNA phenotypes and connects them through the minimum number of changes. The network was initiated with the most frequent phenotype, i.e., I-2. In a first step, all phenotypes that could be explained by a single change with respect to the starting phenotype were added. In a second step, all phenotypes that could be added with a single change to any of the phenotypes already in the network were added. This step was repeated until no single phenotype could be added with just one change. The same step was performed with two changes, three, four, etc., until all phenotypes were added to the network. We followed several assumptions. Each change is independent. Differences equally affecting the two homologous chromosomes were considered as a single change (e.g., a gain/loss of a terminal site or an interstitial site in both chromosomes). Differences affecting one of the two chromosomes, i.e., implying a change from a homozygotic to heterozygotic condition or vice versa were also considered as a single change. Homology of chromosome pairs between different phenotypes was only assumed within species. The frequency of each phenotype in the whole sample (n = 196) is also represented in the network.

## Results

### Chromosome numbers and karyotype features

**Fig 1:**
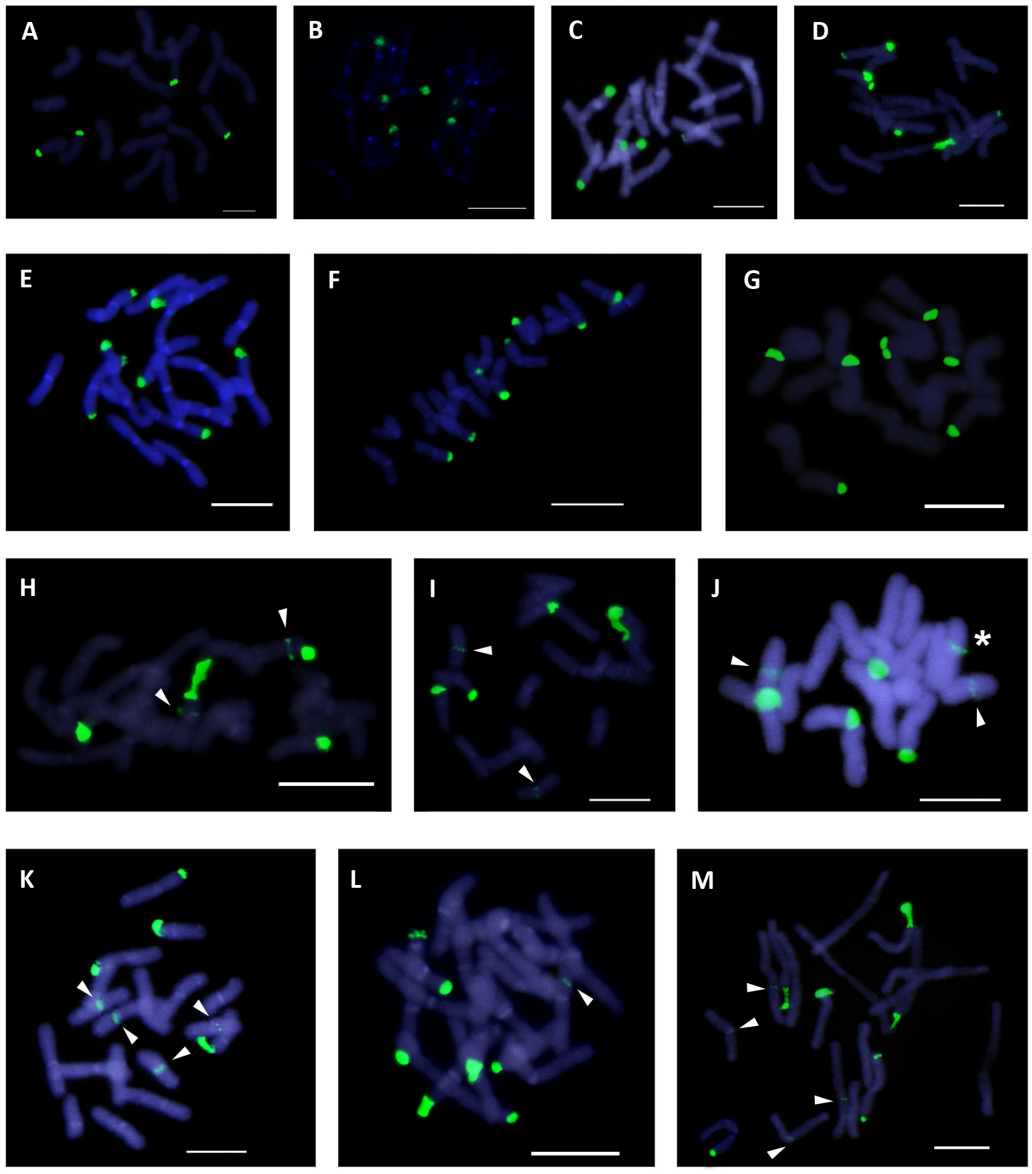
Karyological 45S rDNA site phenotypes in *Anacyclus* assessed by FISH analysis. 45S rDNA sites are shown as green fluorescent signals and the chromosomes are counterstained with 4, 6-diamidino-2-phenylindole (DAPI)-staining (blue colour). (A–G) Class I phenotypes (I-1 –I-7). (H–M) Class II phenotypes (II-1 –II-7). The phenotype II-4 is not shown. Class I phenotypes: (A) I-1, *A*. *linearilobus*. (B) I-2, *A*. *clavatus*. (C) I-3, *A*. *clavatus*. (D) I-4, *A*. *homogamos*. (E) I-5, *A*. *homogamos*. (F) I-6, *A*. *maroccanus*. (G) I-7, *A*. *clavatus*. Class II phenotypes: (H) II-1, *A*. *monanthos*. (I) II-2, *A*. *valentinus*. (J) II-3, *A*. *valentinus*. (K) II-5, *A*. *clavatus*. (L) II-6, *A*. *valentinus*. (M) II-7, *A*. *valentinus*. Chromosomes bearing interstitial 45S rDNA sites are indicated by arrows. The occurrence of extra chromosomes is indicated by an asterisk. Scale bars: 10 μm.

With a single exception, the sporophytic chromosome number 2n = 18 was observed in all *Anacyclus* individuals analysed, in accordance with previous reports ([18], and references therein). The occurrence of the deviant cytotype 2n = 19 was detected in two individuals from one population of *A*. *valentinus* (Fig 1). The extra chromosome was metacentric, and similar in size to the largest chromosome of the complement. This extra chromosome was mitotically stable and may be interpreted either as a trisomy or as a B chromosome.

The karyotypes of *Anacyclus* species comprised metacentric and submetacentric chromosomes of similar size (Fig 1).

### rDNA sites and chromosomal phenotypes

A summary of the results found in the analysis of the number and chromosomal location of the 45S rDNA sites in 196 individuals from 47 populations is shown in Tables 1 and 2, and S2 Table. The number of 45S rDNA sites ranged from four (*A*. *atlanticus*, *A*. *linearilobus*, *A*. *pyrethrum*, and several populations of *A*. *clavatus* and *A*. *valentinus*) to 11 in one individual of *A*. *valentinus* (S2 Table).

**Table 1:** Karyological 45S rDNA phenotypes in *Anacyclus* species assessed by FISH. Each phenotype was defined by the number, chromosomal location and dosage of the 45S rDNA sites. H and HE refer to those sites showing the homomorphic (H) or heteromorphic (HE) dosage.

Karyological phenotypes	No. of sites	Location			
		Terminal		Interstitial	
		Number of sites	Dosage	Number of sites	Dosage
**I-1**	4	4	H	0	-
**I-2**	6	6	H	0	-
**I-3**	5	5	HE	0	-
**I-4**	8	8	H	0	-
**I-5**	8	8	HE	0	-
**I-6**	8	8	H	0	-
**I-7**	7	7	HE	0	-
**II-1**	6	4	H	2^s^	H
**II-2**	6	4	H	2	H
**II-3**	7	4	H	3*	H
**II-4**	8	4	H	2 + 2^s^	H
**II-5**	8	4	H	4	HE
**II-6**	8	7	HE	1	HE
**II-7**	11	7	HE	4	H, HE

^s^ The number of interstitial rDNA sites present on the same chromosomes also bearing terminal sites.

* The occurrence of extra chromosomes bearing an rDNA interstitial site.

**Table 2:** Distribution of rDNA phenotypes and the number of 45S rDNA sites in *Anacyclus* species.

Species	Sample size	Karyological phenotypes	Total no. of rDNA sites
*A*. *atlanticus*	10	I-1	4
*A*. *clavatus*	67	I-1, I-2, I-3, I-7, II-2, II-5	4, 5, 6, 7, 8
*A*. *homogamos*	15	I-2, I-4, I-5	6, 8
*A*. *linearilobus*	4	I-1	4
*A*. *maroccanus*	11	I-6	8
*A*. *monanthos*	10	II-1, II-4	6, 8
*A radiatus*			
subsp. *coronatus*	10	I-2	6
subsp. *radiatus*	12	I-2	6
*A*. *pyrethrum*	9	I-1	4
*A*. *valentinus*	45	I-1, I-2, I-7, II-2, II-3, II-6, II-7	4, 6, 7, 8, 11

**Fig 2:**
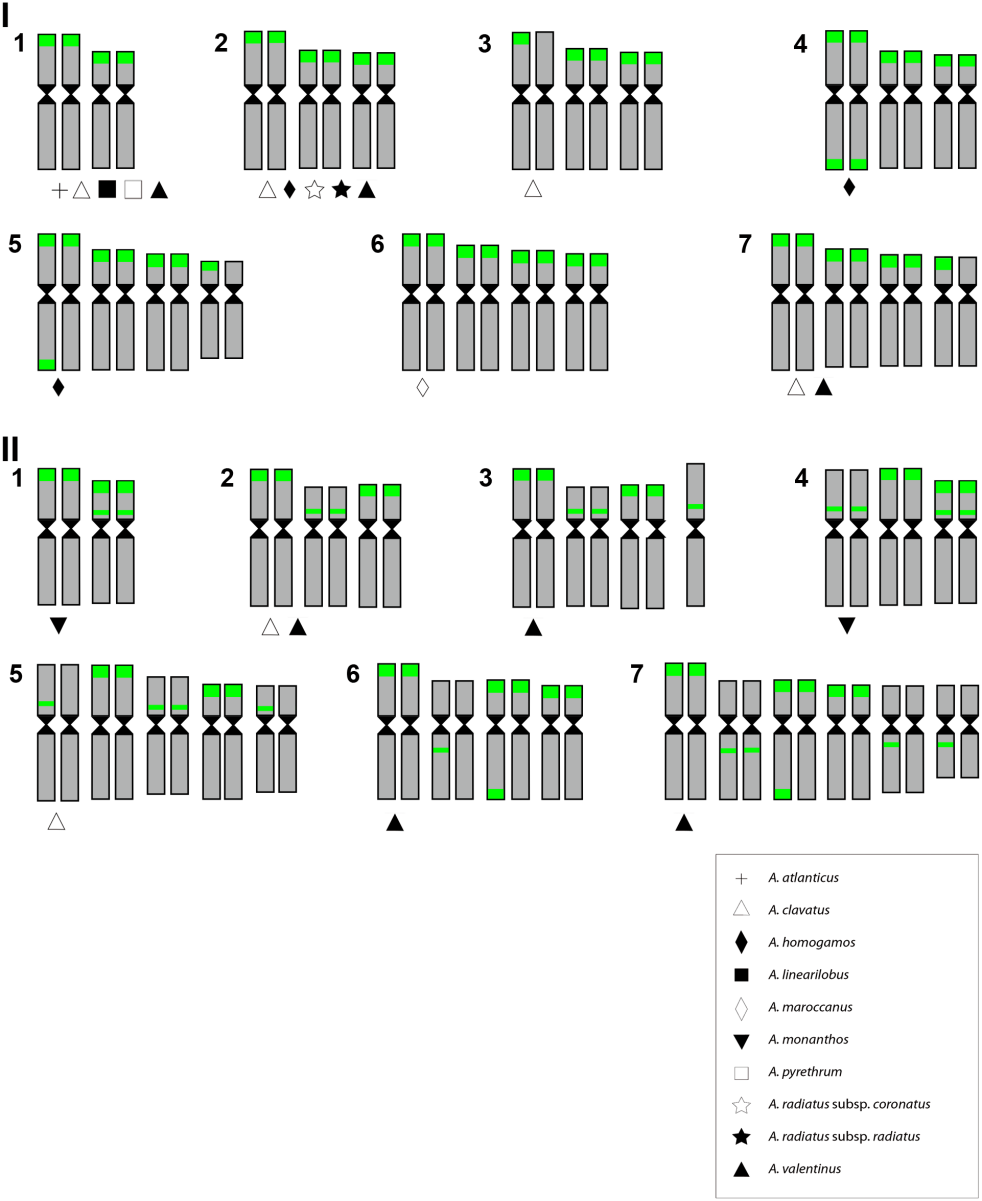
Karyological ribosomal phenotypes identified in *Anacyclus*. The ribosomal phenotypes are described in Table 1. The presence of the ribosomal phenotype in *Anacyclus* species is indicated.

Fourteen karyological ribosomal phenotypes were identified on the basis of site number (in regular and extra chromosomes), chromosomal location (terminal or interstitial), and dosage (homozygosis or heterozygosis) (Tables 1 and 2; Fig 1). These phenotypes were arbitrarily classified into two groups depending on whether interstitial rDNA sites were absent (type I) or present (type II) in the chromosomes. Half of the phenotypes showed heterozygosity for ribosomal sites, specifically at one (I-3, I-7, II-3; Fig 1C, 1G and 1J, respectively and Fig 2), two (I-5, II-5, II-6; Fig 1E, 1K and 1L, respectively and Fig 2) or three loci (II-7; Figs 1M and 2). Phenotypes with heterozygous sites were restricted to three species: *A*. *clavatus*, *A*. *homogamos*, and *A*. *valentinus*. The extra chromosome found in *A*. *valentinus* showed an interstitial 45S rDNA site (rDNA phenotype II-3; Figs 1J and 2).

### Patterns of rDNA variation

The frequency of each rDNA phenotype was skewed towards the I-1 and I-2 types, which were detected in seven out of the nine *Anacyclus* species and together represented 70% of the analysed accessions (Table 2).

A single rDNA phenotype was detected in the perennials *A*. *atlanticus* and *A*. *pyrethrum*, as well as in the annuals *A*. *linearilobus*, *A*. *maroccanus*, and the two subspecies of *A*. *radiatus*. The remainder of the taxa exhibited a more complex pattern of rDNA site variation that included intra-specific and intra-population polymorphisms. Two phenotypes were found in *A*. *monanthos* (II-1, II-4) and three in *A*. *homogamos* (I-2, I-4, I-5). However, the highest variability was recorded in *A*. *clavatus* and *A*. *valentinus* with six and seven phenotypes, respectively.

A significant number of rDNA phenotypes (10 out of 14) were exclusive to a single species, although only one was fixed (Table 2; Fig 2). These include I-3 and II-5 (*A*. *clavatus*), I-4 and I-5 (*A*. *homogamos*), I-6 (*A*. *maroccanus*), II-1 and II-4 (*A*. *monanthos*), II-3, II-6 and II-7 (*A*. *valentinus*). The remaining four rDNA phenotypes were shared by two (I-7, II-2), four (I-2) and five species (I-1; Table 2; Fig 2).

**Fig 3:**
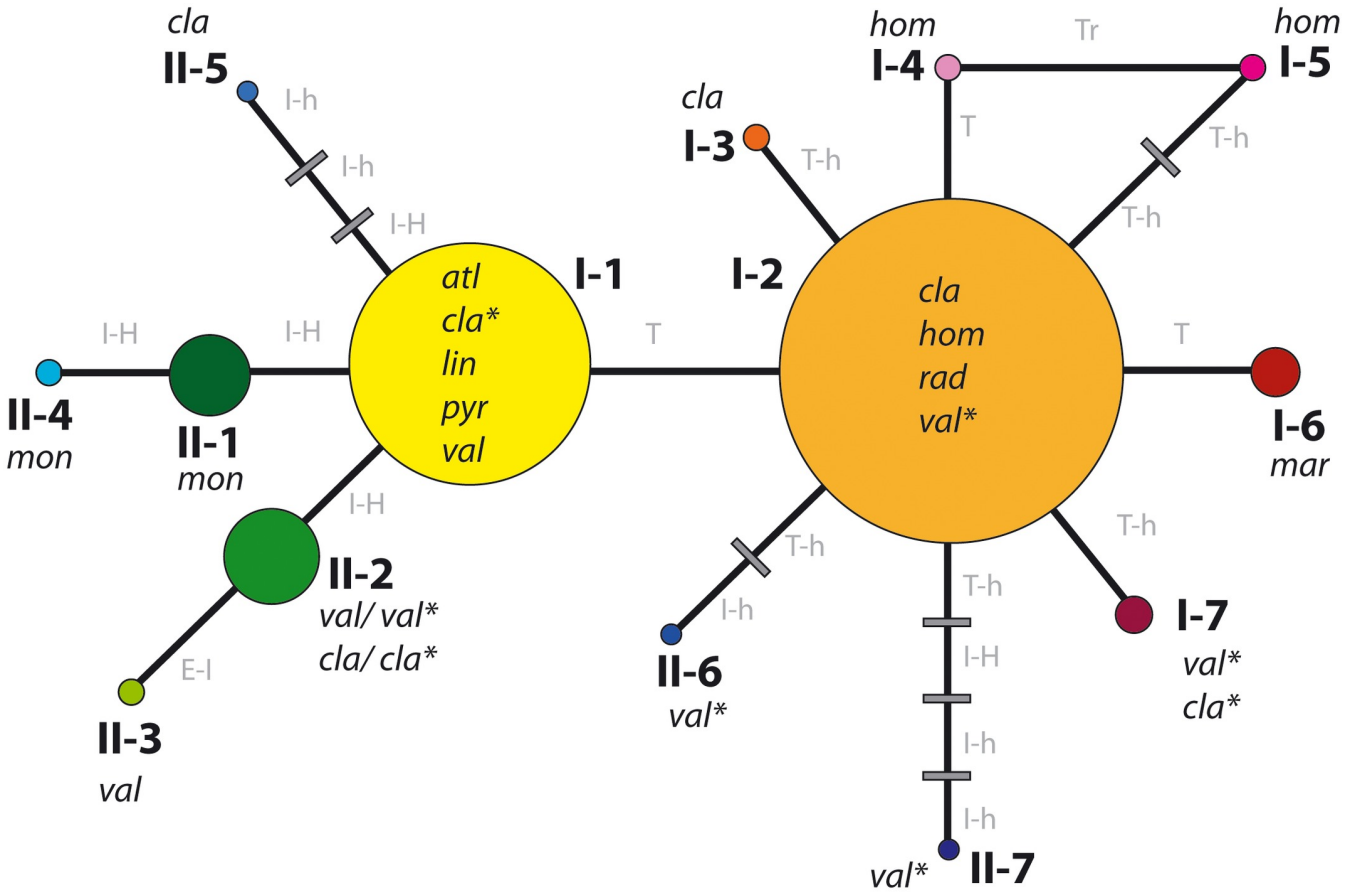
Network connecting fourteen karyological ribosomal phenotypes identified in this study. Each stretch of each branch represents one cytogenetic change (T, terminal site; T-h, heterozygous terminal site; I-H, homozygous interstitial site; I-h, heterozygous interstitial site; E-I, extra chromosome with interstitial site; Tr, translocation). Grey stripes indicate undetected phenotypes. Phenotypes are identified by the symbols used in Table 1 and color-coded circles. Diameter of the circles is proportional to phenotype frequency. Species exhibiting each phenotype are represented by acronyms. Asterisks denote sympatric populations composed of individuals of *A*. *clavatus* and *A*. *valentinus*. See the text for further details.

The hypothesis of rDNA phenotype relationships constructed in the form of phenotype network (Fig 3; S1 Fig) is the most parsimonious way of connecting the 14 phenotypes found by explicit changes, each assumed to be independent. The network is not strictly intended to allow inference of evolutionary relationships but to allow the visualization of the numerical and structural variability of ribosomal sites and the inference of feasible pathways of cytogenetic change. Relationships among phenotypes are discussed below.

## Discussion

### Range of rDNA site variation in *Anacyclus*

The level of rDNA site variation detected in this study is outstanding in the context of angiosperms particularly regarding populations of the same species. The modal number of rDNA sites observed in *Anacyclus* (six) deviates from two and four, which are the most frequent for diploid karyotypes across angiosperms [5]. However, unusually higher number of rDNA sites (12–20) has been documented in at least another diploid genus of Asteraceae, i.e. *Cheirolophus* ([28]; http://www.plantrdnadatabase.com, accessed 18^th^ July 2017).

Heterogeneity on the number of rDNA signals within a species (and even within a population) has been indicated in *Tanacetum* [29], a genus phylogenetically closely related to *Anacyclus*. However, and in contrast with *Anacyclus*, most of this intraspecific variation refers to taxa with odd, high ploidy or aneuploid levels [29], suggesting the occurrence of different underlying genetic causes in the variation of rDNA gene site number.

The range of 45S rDNA site numbers found in *Anacyclus* in this study (4 to 11) is also larger than previous reports within this genus (4 to 6 sites) [18, 19]. Several reasons may account for or contribute to this discrepancy. First, the different techniques used by those studies, which revealed only the active rDNA sites (NOR) in contrast to our molecular cytogenetic approach, may explain the higher number of rDNA sites found in ours. In addition, the large sample size in our study (196 individuals) has probably allowed a more accurate assessment of the levels of intra-specific variation than in previous works. Lastly, sampling of interspecific sympatric populations, where ongoing hybridisation processes may occur, might have revealed a dynamic turnover of rDNA site number associated with genomic rearrangements.

### Relationships between ribosomal phenotypes

The phenotype network allows us to hypothesize on the ancestral conditions and discuss the causes for the least frequent phenotypes (Fig 3; S1 Fig). The internal position in the network, the high frequency of occurrence among species, and the single cytogenetic change separating phenotypes I-1 and I-2 suggest that these are ancestral in *Anacyclus*. Additional evidence for the ancestral condition of phenotype I-1 is its presence in *A*. *atlanticus*, the earliest diverging species of the genus ([30], as *Heliocauta atlantica*). In contrast, the tip phenotypes in the network are the least frequent in our sampling and the most restricted to species and geographic areas, suggesting a derived origin. The early divergent condition of the two most frequent phenotypes could be further explored by ancestral character-state reconstruction upon a solid molecular phylogeny. However, this is currently precluded by the availability of such phylogeny and the simplicity of the models of evolutionary change for characters other than DNA sequences.

Most connections between rDNA phenotypes required one or two changes. The exceptions were the tip phenotypes II-5 and II-7, which required three and four cytogenetic events from phenotypes I-1 and I-2, respectively, to originate under the assumption of a stepwise cytogenetic evolution. The existence of such *missing* phenotypes along the branches leading to phenotypes II-5 and II-7 may suggests that we did not sample all rDNA phenotypes present in *A*. *clavatus* and *A*. *valentinus* (where phenotypes II-5 and II-7 occur), the past existence of now extinct taxa bearing the missing phenotypes, or that the notable genomic instability detected in these species caused a rapid acceleration in the rate of rDNA site-number evolution.

### Mechanisms for rDNA site-number variation

rDNA site-number variation in *Anacyclus* is complex as it involves changes in (i) the number of homozygous 45S rDNA loci, (ii) the occurrence of interstitial sites, (iii) the presence of minor sites in accessory chromosomes, and (iv) the degree of heterozygosity in half of the reported 45S rDNA phenotypes. It is likely that various cytogenetic and molecular mechanisms underlie this rDNA dynamism. However, it is difficult to disentangle the differential contribution of the processes associated with it. This is further complicated by the fact that *Anacyclus* shows very similar karyotypes based on gross chromosomal morphology [18] and accordingly, genome rearrangements are difficult to infer in mitotic stages. In any case, three possible causes can be mentioned.

In angiosperms, the preferential localization of 45S rDNA loci in the terminal positions of chromosomes is the main argument supporting the idea that heterologous recombination can drive rDNA mobility [5, 31]. In *Anacyclus*, both the conserved location of heterochromatin in the proximal regions and its variable presence in the interstitial and terminal regions of chromosomes have been reported [18]. These observations are in agreement with the potential role of non-homologous recombination in the 45S rDNA turnover in this genus, but are not conclusive.

Activity of mobile elements (retrotransposons) that accumulate in the vicinity of rDNA loci has been suggested to be associated with rDNA dynamism [32–38]. Preliminary results in four species in *Anacyclus* indicate that approximately half of their genomes are composed of transposable elements (Vitales et al., pers. comm.).

A third potential cause stems from the observation of high levels of 45S rDNA site-number heterozygosity in populations of *A*. *homogamos*, *A*. *clavatus* and *A*. *valentinus* in sympatry with congeners. It is possible that a larger sample size focusing on non-overlapping areas might reveal additional homozygous rDNA phenotypes. But with the available data, it can be hypothesized that rDNA heterozygosity in these species is associated with hybridisation. Interestingly, heterozygosity in rDNA site-number has also been observed in diploid hybrid species of *Argyranthemum* (Asteraceae) by Borgen et al. [39].

### Implications of rDNA site-number diversity

A previous study has suggested that an increase in the number of nucleolar chromosomes has occurred during the evolution of *Anacyclus* [18]. Specifically, these authors speculated that the perennial state is ancestral in the genus, and that the transition from the perennial to the annual life cycle was accompanied by karyotypic changes leading to an increase in 45S rDNA sites and thereby, an increase in rRNA gene copy numbers.

Our results show that there is no clear association between the number of 45S rDNA loci and the life cycle in *Anacyclus*. For instance, two 45S rDNA loci have been found both in the annual species *A*. *clavatus*, *A*. *valentinus*, and *A*. *linearilobus*, and in the perennials *A*. *atlanticus* and *A*. *pyrethrum*.

In addition, our observations on the number and intensity of the FISH signals can neither support nor reject the predictions of Schweizer and Ehrendorfer [18] that the increase (or decrease) in nucleolar chromosomes may be directly related to a change in the overall rRNA gene copies, since standard FISH cannot be used as an experimental proxy for this evaluation. Furthermore, it has been reported that the number of rRNA copies is apparently independent from the number of NOR loci when comparing homoploid individuals from a single species [40], or conspecific plants showing different ploidy levels [41].

Finally, 45S rDNA loci have been reported as fragile DNA sites that are prone to chromosomal lesions and hotspots of genome rearrangements and genetic instabilities [42–44]. On this basis, it could be hypothesized that, all things being equal, an increase in the number of ribosomal sites might raise the likelihood of cytogenetic abnormalities, compromising the cellular fitness of individuals and microevolutionary processes. However, no chromosome gaps or breakpoints were observed in this study, and the mitotic cycle showed no cytogenetic irregularities at any of the observed stages. Additional FISH studies during meiotic microsporogenesis are required to further corroborate the lack of association between the number of 45S rDNA sites and cytological abnormalities.

### Patterns in rDNA site-number variation

**Fig 4:**
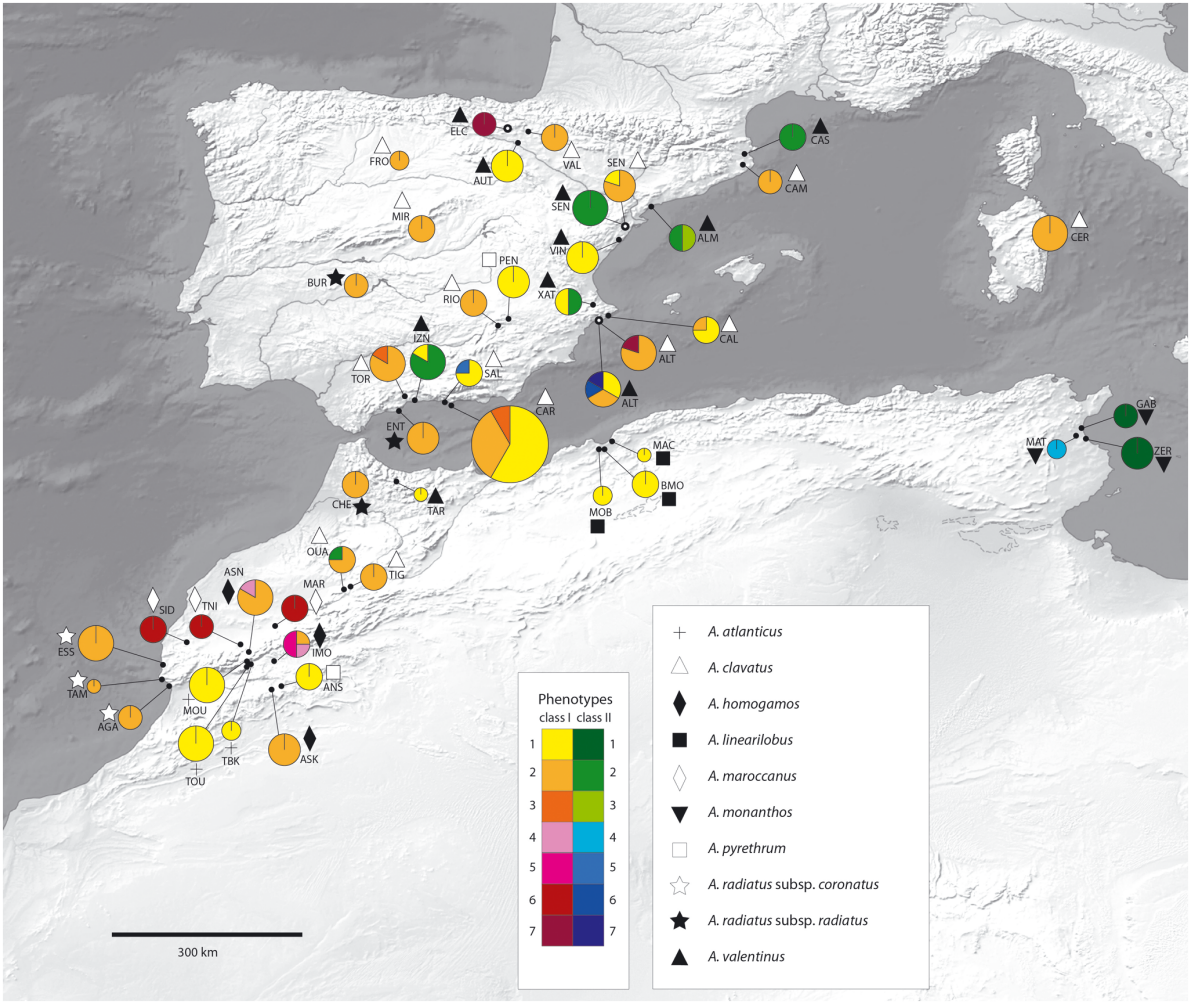
Geographic distribution of karyological ribosomal phenotypes in *Anacyclus*. Circle size is proportional to the sampling in each population. Sympatric populations of *A*. *clavatus* and *A*. *valentinus* are indicated by an empty dot. See Fig 2 for phenotype descriptions. The map is made with Natural Earth. Free vector and raster map data @naturalearthdata.com.

The distribution of rDNA site variation across species and geographical areas is not even. Although convergent evolution cannot be excluded to explain the sharing of the same phenotypes among different species, there are some taxonomic patterns such as two species showing exclusive phenotypes (*A*. *maroccanus*, *A*. *monanthos*; Figs 3 and 4) whereas others exhibiting only one, although not exclusive, phenotype. For instance, *A*. *atlanticus*, *A*. *linearilobus* and *A*. *pyrethrum* share phenotype I-1 whereas *A*. *radiatus* shares I-2 with three other species, *A*. *homogamos*, *A*. *clavatus*, *A*. *valentinus* (Figs 3 and 4). However, for the latter three species a clear taxonomic pattern is not seen although regularities are apparent, e.g., phenotype I-1 is more frequent in *A*. *valentinus* whereas I-2 is more frequent in *A*. *clavatus* and *A*. *homogamos* (Fig 4).

We have not found any relationship between the variation in rDNA phenotypes and major ecological conditions. The two non-weedy species (*A*. *atlanticus* and *A*. *linearilobus*) presented just one phenotype (I-1). But the remaining seven species, all growing in the same anthropogenic habitats such as roadsides, railroads, and in general open disturbed places, represented different situations for rDNA phenotypes. Some of them presented the highest number but others just showed one (e.g., *A*. *maroccanus* and *A*. *radiatus)*. Climatic differences exist in the ranges of some species (i.e., *A*. *radiatus* occurs in places with Atlantic climate influence whereas *A*. *valentinus*, *A*. *monanthos*, and *A*. *linearilobus* grow in Mediterranean climate areas). However, these major differences do not show association with variation of rDNA site number and rDNA phenotypes.

Why is rDNA site variation higher in *Anacyclus clavatus*, *A*. *valentinus*, and *A*. *homogamos*? Several non-mutually exclusive hypotheses may underlie this pattern. First, the greatest variation could just coincide with the species with higher sample sizes. A Pearson correlation analysis shows that overall the number of individuals analyzed significantly correlates with the number of observed phenotypes (r = 0.487, p<0.001). Thus, it could not be discarded that intensive sampling may reveal additional variation in other narrowly distributed species (e.g., *A*. *atlanticus*, *A*. *linearilobus*). Second, it could be argued that the three aforementioned species have large ranges and thus their variation could be associated with some degree of ecogeographic differentiation (S2 Fig). The fact that other species with large ranges, such as *A*. *radiatus*, *A*. *monanthos* and to a lesser degree *A*. *pyrethrum*, exhibit one or at most two 45S rDNA phenotypes neither rejects nor supports this possibility because a larger range need not lead to differentiation. On the other hand, if this was a significant cause one would expect some kind of geographic structuring of rDNA phenotype variations within those species with wider ranges, but such is not the case.

The third possibility, which we consider the most likely or influential, is that the remarkable number of phenotypes in *A*. *homogamos* and particularly *A*. *clavatus* and *A*. *valentinus* is likely to be associated with contemporary hybridisation events along different parts of their ranges. A marked variability in inflorescence phenotypes has been observed in sympatric populations, including the occurrence of intermediate phenotypes (e.g., shortly-rayed heads compared to conspicuously-rayed heads in *A*. *clavatus* and discoid in *A*. *valentinus*; [20]; I. Álvarez, pers. obs.). These intermediate phenotypes are routinely obtained in synthetic crosses [19], whereas they are very rarely observed in non-overlapping areas of the two species. All tested species in *Anacyclus* are obligate outcrossers [19], the large distribution areas of *A*. *clavatus* and *A*. *valentinus* largely overlap, and the habitat in which they grow is the same (S2 Fig) which facilitates interspecific hybridisation.

Of the eight phenotypes occupying the terminal positions in the network, three correspond to sympatric populations (I-7, II-6, II-7) and three to overlapping areas (I-3, II-3, II-5; Fig 4). To distinguish changes due to hybridisation from those due to other processes over evolutionary time, it would be necessary to clarify whether, in addition to contemporary hybridisation there were older hybridisation events in *Anacyclus*, e.g., resulting in the origin of species like *A*. *valentinus*, as hypothesized by Humphries [19, 20]. This should be addressed in a future phylogenetic study.

## Conclusions

The 45S rDNA site number, their chromosomal distribution and structure have been assessed in a significant sample of seed plants, which usually exhibit rather consistent features, except for polyploid wild plants. In contrast, the level of rDNA site-number variation detected in *Anacyclus* is outstanding. Specifically, it spans a large part of the range of variation found per diploid complement (i.e., corrected for polyploidy) across all angiosperms. Interestingly, the distribution of rDNA site-number variation, their chromosomal location and dosage are not even across species and geographical areas. Apparently, there are no clear relationships between environment, floral morphology, breeding system and phylogenetic position of the species and the number of rDNA sites and rDNA phenotypes. Nevertheless, some patterns are seemingly consistent with species boundaries (*A*. *atlanticus*, *A*. *pyrethrum*, *A*. *linearilobus*, *A*. *maroccanus* and *A*. *radiatus*). However, a more complex pattern of rDNA variation that included intra-specific and intra-population polymorphisms was recorded in *A*. *homogamos*, *A*. *clavatus* and *A*. *valentinus*, three weedy species showing large and overlapping distribution ranges. It is likely that part of the cytogenetic changes and inferred dynamism explaining the differences among the phenotypes found in these species have been triggered by genomic rearrangements resulting from contemporary hybridisation.

## Supporting information

S1 Table Accessions of *Anacyclus* species analysed by FISH, with their origins and sample size. (DOC)

S2 Table Distribution of rDNA phenotypes and the number of 45S rDNA sites in the populations of *Anacyclus* species analysed. (DOC)

S1 Fig Network connecting fourteen ribosomal phenotypes identified in this study in Fig 3 with schematic representation of phenotypes as in Fig 2. Each stretch of each branch represents one cytogenetic change (T, T-h, I-H, I-h, E-I and Tr). Grey stripes indicate undetected phenotypes. Phenotypes are described in Table 1 and Fig 2. Species exhibiting each phenotype are represented by the symbols used in Fig 2. Asterisks denote sympatric populations composed of individuals of *A*. *clavatus* and *A*. *valentinus*. See the text for details.
(TIFF)

S2 Fig Distribution ranges of the *Anacyclus* species. Geographically isolated areas are indicated by symbols. Made with Natural Earth. Free vector and raster map data @ naturalearthdata.com.
(TIFF)
