# Supplementary material for: High and uneven levels of 45S rDNA site-number variation across wild populations of a diploid plant genus (Anacyclus, Asteraceae)
Source: PLoS One. 2017 Oct 31;12(10):e0187131. doi: 10.1371/journal.pone.0187131 (PMC5663423; doi:10.1371/journal.pone.0187131)
Supplement: S1 Table — (DOC) [file pone.0187131.s001.doc]

S1 Table. Accessions of *Anacyclus* species analysed by FISH, with their origins and sample size.

| Species | Accession | Population code | Sample size |
| --- | --- | --- | --- |
| *A. atlanticus Litard. & Maire* | Morocco, High Atlas, Jebel Toubkal, near refuge “Les Mouflons”, 31°3’44.69”N 7°56’16.4”W, 3206 m, 13-IX-2016, *Álvarez 2348* | Mou | 4 |
|  | Morocco, High Atlas, Jebel Toubkal, 31°3’38.72”N 7°55’41.87”W, 3721 m, 14-IX-2016, *Álvarez 2350* | Tbk | 2 |
|  | Morocco, High Atlas, Jebel Toubkal, 31°3’44.13”N 7°56’3.62”W, 3515 m, 14-IX-2016, *Álvarez 2349* | Tou | 4 |
| *A. clavatus* (Desf.) Pers | Spain, Alicante, Altea, L’Horta, 38°36’18.62”N 0°2’57.97”W, 25 m, 24-V-2008, *Medina 4434* | Alt | 5 |
|  | Spain, Alicante, Calpe, 38°38’23”N 0°4’31.26”E, 50 m, 27-VI-2009, *Álvarez 2022* | Cal | 4 |
|  | Spain, Girona, Camallera, 42°7’27.2”N 2°57’23.5”E, 100 m, 29-VI-2009, *Álvarez 2050* | Cam | 3 |
|  | Spain, Granada, Carchuna, 36°41’49”N 3°27’33”W, 13 m, 27-IV-2011, *Agudo 1* | Car | 12 |
|  | Italy, Sardinia, Nuoro, Dorgali, Serra Orrios, 40°19’59.9”N 9°31’53.5”E, 175 m, 28-VI-2010, *Medina 5110* | Cer | 6 |
|  | Spain, Palencia, Frómista, 42°16’3.75”N 4°24’19.23”W, 780 m, 29-VIII-2009, *Álvarez 2073* | Fro | 2 |
|  | Spain, Madrid, Miraflores de la Sierra, Las Huelgas, 40°47’36.45”N 3°43’46.97”W, 883 m, 22-X-2011, *Álvarez 2173* | Mir | 4 |
|  | Morocco, near Ouaoumana, 32°43’1.9”N 5°47’56.5”W, 762 m, 5-VI-2012, *Agudo 47* | Oua | 4 |
|  | Spain, Albacete, Riópar, 38°30’16.46”N 2°26’52.9”W, 1135 m, 9-VIII-2013, *Álvarez 2333* | Rio | 4 |
|  | Spain, Granada, Salobreña, 36°43’30.1”N 3°34’50.4”W, 0 m, 30-III-2011, *Álvarez 2135* | Sal | 4 |
|  | Spain, Tarragona, La Sénia, 40°37’55.2”N 0°16’52”E, 380 m, 28-VI-2009, *Álvarez 2034* | Sen | 5 |
|  | Morocco, near Tighassaline, 32°45’23.4”N 5°40’30.9”W, 871 m, 5-VI-2012, *Agudo 49* | Tig | 4 |
|  | Spain, Málaga, Torcal de Antequera, 36°57’46.4”N 4°30’48.8”W, 947 m, 28-III-2011, *Álvarez 2123* | Tor | 6 |
|  | Spain, Navarra, Valtierra, 42°12’8”N 1°38’43”W, 260 m, 18-VIII-2009, *Álvarez 2069* | Val | 4 |
| *A. homogamos* (Maire) Humphries | Morocco, High Atlas, near Askaun, 30°44’2.9”N 7°47’51.2”W, 1943 m, 9-VI-2009, *Quintanar 3505* | Ask | 5 |
|  | Morocco, Middle Atlas, Asni, 31°15’4”N 7°58’40”W, 1160 m, 24-V-2010, *Álvarez 2115* | Asn | 6 |
|  | Morocco, High Atlas, near Imouzzer, 31°19’55”N 7°24’32”W, 2224 m, 13-VI-2009, *Gonzalo 1275* | Imo | 4 |
| *A. linearilobus* Boiss. & Reuter | Algeria, Oran, Bomo beach, near Bou-Sfer, 35°44’52.53”N 0°49’56.53”W, 19 m, dunes, 23-V-2016, *Álvarez 2340* | Bmo | 4 |
|  | Algeria, Mers El Hadjadj, La Macta beach, 35°47’22.65”N 0°9’9.84”W, 2 m, dunes, 25-V-2016, *Álvarez 2345* | Mac | 1 |
|  | Algeria, Oran, Bomo beach, near Bou-Sfer, 35°45’43.48”N 0°49’46.68”W, 4 m, dunes, 23-V-2016, *Álvarez 2339* | Mob | 2 |
| *A. maroccanus* (Ball) Ball | Morocco, between Marrakech and El Kelaa des Sraghna, 31°53’31”N 7°26’23”W, 570 m, 4-VI-2012, *Agudo 33* | Mar | 4 |
|  | Morocco, road from Marrakech to Essaouira, near Sidi Mokhtar, 31°35’16.86”N 9°4’17.52”W, 373 m, 21-V-2010, *Álvarez 2099* | Sid | 4 |
|  | Morocco, near Marrakech, Tnine-des-Oudaya, 31°37’55.66”N 8°15’10.67”W, 380m, 21-V-2010, *Álvarez 2097* | Tni | 3 |
| *A. monanthos* (L.) Thell. | Tunisia, Gabès, 33°53’11”N 10°1’6.2”E, 50 m, 23-III-2009, *Aedo 16194* | Gab | 3 |
|  | Tunisia, Matmata, 33°44’33”N 10°0’56”E, 100 m, 23-III-2009, *Aedo 16233* | Mat | 2 |
|  | Tunisia, Zerkine, 33°45’37.23”N 10°15’59”E, 20 m, 24-III-2009, *Aedo 16310* | Zer | 5 |
| *A. radiatus* Loisel*.* |  |  |  |
| subsp. *coronatus* | Morocco, road between Imessouane and Agadir, 30°46’20.07”N 9°48’12.09”W, 286 m, 22-V-2010, *Álvarez 2107* | Aga | 3 |
|  | Morocco, Essaouira, 31°32’1.55”N 9°44’37.83”W, 5 m, dunes, 22-V-2010, *Álvarez 2105* | Ess | 6 |
|  | Morocco, between Tamri and Amesnaz, 30°39’5.21”N 9°53’12.01”W, 4 m, dunes, 22-V-2010, *Álvarez 2108* | Tam | 1 |
| subsp. *radiatus* (Murb.) Humphries | Spain, Cáceres, río Búrdalo, 39°5’42”N 6°0’51”W, 261 m, 9-IV-2012, *Álvarez 2177* | Bur | 3 |
|  | Morocco, near Chefchaouen, 35°20’15.9”N 5°21’46.5”W, 640 m, 10-IV-2012, *Álvarez 2180* | Che | 4 |
|  | Spain, Málaga, Entrerríos, 36°33’6.5”N 4°41’47.3”W, 35 m, *Álvarez 2138* | Ent | 5 |
| *A. pyrethrum* (L.) Link | Spain, Albacete, Sierra de Alcaraz, Peñascosa, 38°38’25”N 2°22’41.26”W, 1276 m, 9-VIII-2013, *Álvarez 2334* | Pen | 5 |
|  | Morocco, High Atlas, between Askaun y Ansal, 30°44’37.4”N 7°42’24.8”W, 2337 m, 10-VI-2009, *Quintanar 3531* | Ans | 4 |
| *A. valentinus* L. | Spain, Tarragona, L’Almetla de Mar, 40°56’6.5”N 0°51’24.4”E, 1 m, 28-VI-2009, *Álvarez 2038* | Alm | 4 |
|  | Spain, Alicante, Altea, L’Horta, 38°36’18.62”N 0°2’57.97”W, 25 m, 24-V-2008, *Medina 4435* | Alt | 6 |
|  | Spain, La Rioja, Autol, 42°12’47”N 2°0’21”W, 450 m, 17-VIII-2009, *Álvarez 2067* | Aut | 5 |
|  | Spain, Girona, Castelló d’Empuries, 42°15’47.2”N 3°7’45.5”W, 0 m, *Álvarez 2059* | Cas | 5 |
|  | Spain, Álava, Elciego, 42°32’1.61”N 2°21.74”W, 485 m, 17-VIII-2009, *Álvarez 2065* | Elc | 3 |
|  | Spain, Málaga, Iznate, 36°46’35”N 4°10’45”W, 285 m, 30-III-2011, *Álvarez 2137* | Izn | 6 |
|  | Spain, Tarragona, La Sénia, 40°37’55.2”N 0°16’52”E, 380 m, 28-VI-2009, *Álvarez 2033* | Sen | 6 |
|  | Morocco, near Targuist, 34°57’57”N 4°22’40”W, 1328 m, 18-VI-2008, *Santos 1072* | Tar | 1 |
|  | Spain, Castellón, Vinaròs, 40°28’54.6”N 0°28’20.1”E, 8 m, *Álvarez 2032* | Vin | 5 |
|  | Spain, Valencia, Xàtiva, 38°59’45.2”N 0°30’14”W, 95 m, 27-VI-2009, *Álvarez 2025* | Xat | 4 |
