# Supplementary material for: High and uneven levels of 45S rDNA site-number variation across wild populations of a diploid plant genus (Anacyclus, Asteraceae)
Source: PLoS One. 2017 Oct 31;12(10):e0187131. doi: 10.1371/journal.pone.0187131 (PMC5663423; doi:10.1371/journal.pone.0187131)
Supplement: S2 Table — (DOC) [file pone.0187131.s002.doc]

S2 Table. Distribution of rDNA phenotypes and the number of 45S rDNA sites in the populations of *Anacyclus* species analyzed.

| Species | Population code | Karyological ribosomal phenotypes | No. of rDNA sites |
| --- | --- | --- | --- |
| A. atlanticus | Mou | I-1 | 4 |
|  | Tbk | I-1 | 4 |
|  | Tou | I-1 | 4 |
|  |  |  |  |
| A. clavatus | Alt | I-2, I-7 | 6, 7 |
|  | Cal | I-1, I-2 | 4, 6 |
|  | Cam | I-2 | 6 |
|  | Car | I-1, I-2, I-3 | 4, 5, 6 |
|  | Cer | I-2 | 6 |
|  | Fro | I-2 | 6 |
|  | Mir | I-2 | 6 |
|  | Oua | I-2, II-2 | 6 |
|  | Rio | I-2 | 6 |
|  | Sal | I-1, II-5 | 4, 8 |
|  | Sen | I-1, I-2 | 4, 6 |
|  | Tig | I-2 | 6 |
|  | Tor | I-2, I-3 | 4, 5 |
|  | Val | I-2 | 6 |
|  |  |  |  |
| A. homogamos | Ask | I-2 | 6 |
|  | Asn | I-2, I-4 | 6, 8 |
|  | Imo | I-2, I-4, I-5 | 6, 8 |
|  |  |  |  |
| A. linearilobus | Bmo | I-1 | 4 |
|  | Mac | I-1 | 4 |
|  | Mob | I-1 | 4 |
|  |  |  |  |
| A. maroccanus | Mar | I-6 | 8 |
|  | Sid | I-6 | 8 |
|  | Tni | I-6 | 8 |
|  |  |  |  |
| A. monanthos | Gab | II-1 | 6 |
|  | Mat | II-4 | 8 |
|  | Zer | II-1 | 6 |
|  |  |  |  |
| A. radiatus |  |  |  |
| subsp. *coronatus* | Aga | I-2 | 6 |
|  | Ess | I-2 | 6 |
|  | Tam | I-2 | 6 |
|  |  |  |  |
| subsp. *radiatus* | Bur | I-2 | 6 |
|  | Che | I-2 | 6 |
|  | Ent | I-2 | 6 |
|  |  |  |  |
| A. pyrethrum | Pen | I-1 | 4 |
|  | Ans | I-1 | 4 |
|  |  |  |  |
| A. valentinus | Alm | II-2, II-3 | 6, 7 |
|  | Alt | I-1, I-2, II-6, II-7 | 4, 6, 8, 11 |
|  | Aut | I-1 | 4 |
|  | Cas | II-2 | 6 |
|  | Elc | I-7 | 7 |
|  | Izn | I-1, II-2 | 4, 6 |
|  | Sen | II-2 | 6 |
|  | Tar | I-1 | 4 |
|  | Vin | I-1 | 4 |
|  | Xat | I-1, II-2 | 4, 6 |
|  |  |  |  |
