# Supplementary figures and images for: High and uneven levels of 45S rDNA site-number variation across wild populations of a diploid plant genus (Anacyclus, Asteraceae)
Source: PLoS One. 2017 Oct 31;12(10):e0187131. doi: 10.1371/journal.pone.0187131 (PMC5663423; doi:10.1371/journal.pone.0187131)

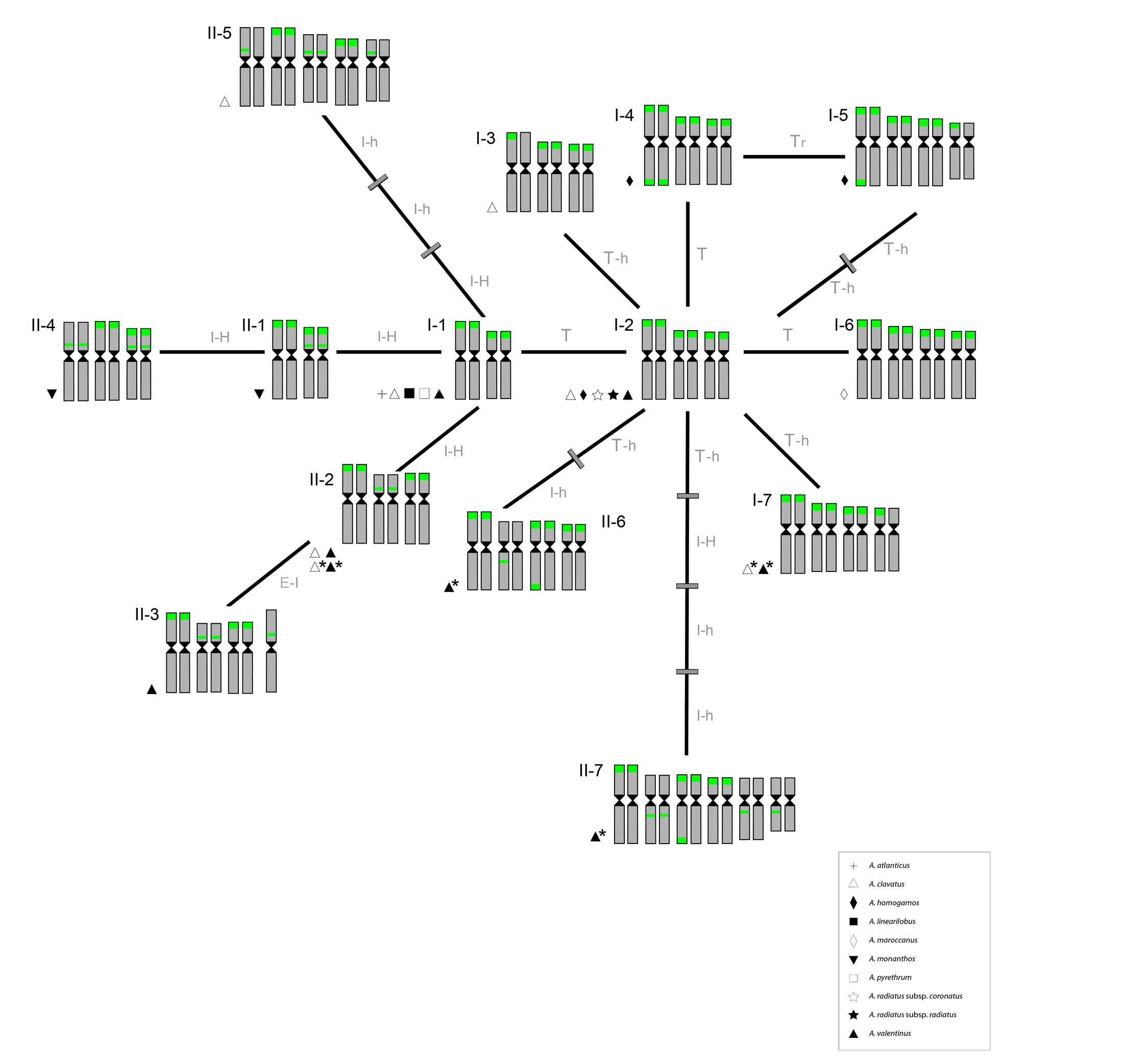

Supplement: S1 Fig — Each stretch of each branch represents one cytogenetic change (T, T-h, I-H, I-h, E-I and Tr). Grey stripes indicate undetected phenotypes. Phenotypes are described in Table 1 and Fig 2. Species exhibiting each phenotype are represented by the symbols used in Fig 2. Asterisks denote sympatric populations composed of individuals of A. clavatus and A. valentinus. See the text for details. (TIFF) [file pone.0187131.s003.tiff]

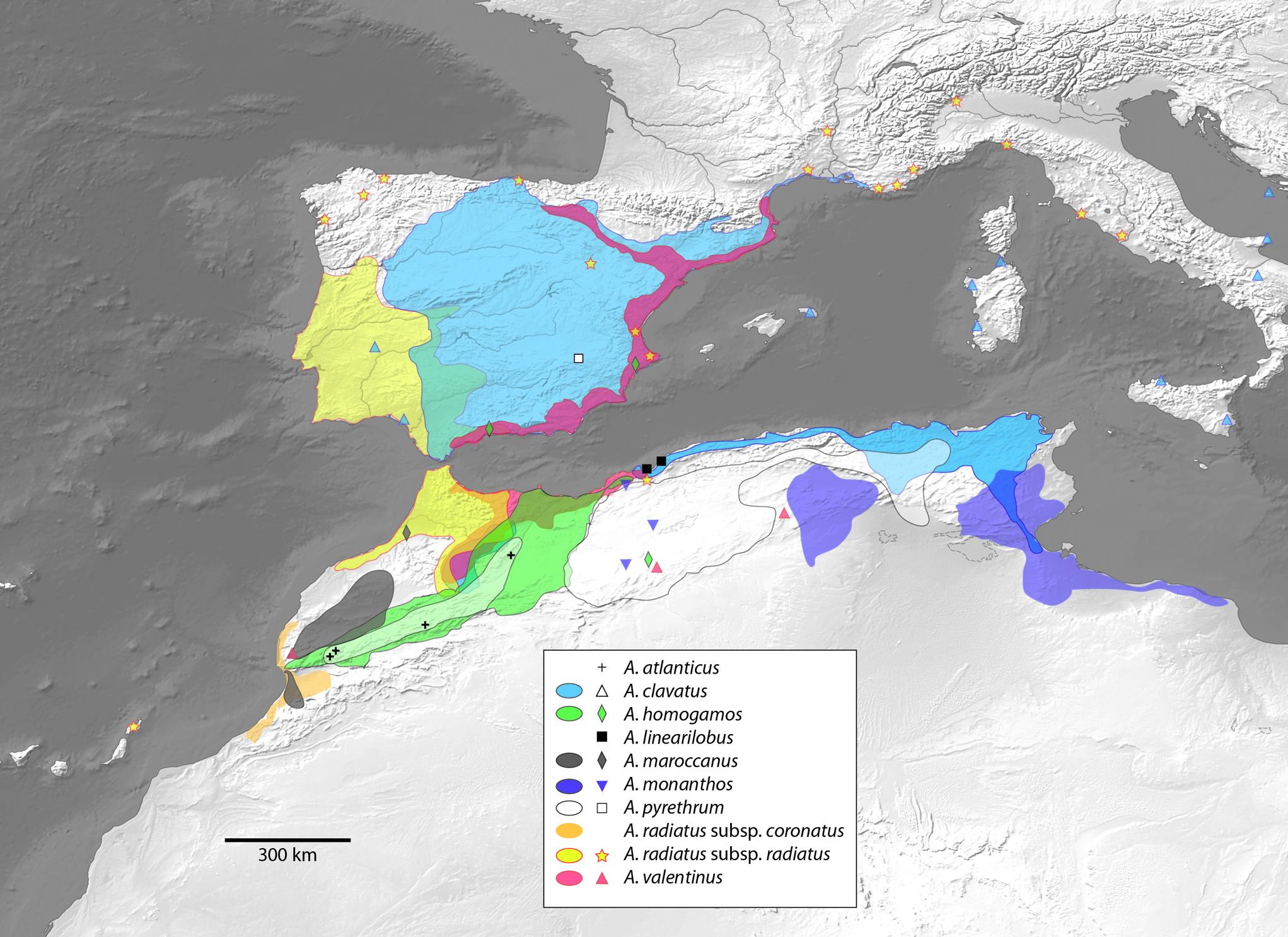

Supplement: S2 Fig — Geographically isolated areas are indicated by symbols. Made with Natural Earth. Free vector and raster map data @ naturalearthdata.com. (TIFF) [file pone.0187131.s004.tiff]
